# Rising Demand for Fetoscopic Laser Therapy for Twin-to-Twin Transfusion Syndrome: Trends, Maternal Age Insights, and Future Challenges in Germany

**DOI:** 10.3390/jcm14134476

**Published:** 2025-06-24

**Authors:** Anna Dionysopoulou, Kathrin Stewen, Yaman Degirmenci, Lina Judit Schiestl, Konstantin Hofmann, Annette Hasenburg, Roxana Schwab

**Affiliations:** Department of Obstetrics and Gynecology, University Medical Center of the Johannes Gutenberg University Mainz, 55131 Mainz, Germany; kathrin.stewen@unimedizin-mainz.de (K.S.); yaman.degirmenci@unimedizin-mainz.de (Y.D.); lina.schiestl@unimedizin-mainz.de (L.J.S.); konstantin.hofmann@unimedizin-mainz.de (K.H.); annette.hasenburg@unimedizin-mainz.de (A.H.); roxana.schwab@unimedizin-mainz.de (R.S.)

**Keywords:** twin-to-twin-transfusion syndrome, monochorionic twin pregnancy, fetoscopic laser surgery, serial amniondrainage, future challenges, healthcare systems

## Abstract

**Background/Objectives:** The twin-to twin transfusion syndrome (TTTS) is the most common complication of monochorionic twin pregnancies. Fetal laser therapy (FLT) and serial amniondrainage (SAD) have been used as treatment options for TTTS. This study examines how the management of TTTS in Germany has evolved in the past years and addresses future patient needs and potential challenges for healthcare providers and healthcare systems. **Methods:** The number of TTTS-related interventions between the years 2005 and 2021 were extracted from the German Federal Statistical Office. The trajectory of FLT and SAD procedures over the study period was analyzed. The historical data were used to make projections for future years and address future FLT surgical needs. Further, we aimed to determine age-related influences in monochorionic twin pregnancies requiring FLT. **Results:** A statistically significant increase in the number of FLT surgeries and a noteworthy decline in the number of SAD procedures with respect to both the number of deliveries per year and the number of multiple pregnancies per year were noted. For the first time, we showed that the percentage of multiple pregnancies requiring FLT was significantly higher in younger mothers under 25 years of age, than in all other age groups. **Conclusions:** For the moment, FLT poses the only direct and causative treatment of TTTS. The results of our analysis reveal an increasing demand for FLT surgeries for future years. We highlight the need to train more maternal–fetal medicine specialists to be able to perform the procedure safely and to allocate resources efficiently to accommodate the rising number of cases.

## 1. Introduction

The estimated prevalence of monochorionic twins is around 1:250 to 1:400 naturally conceived pregnancies. They account for 2/3 of monozygotic and 20% of all twin pregnancies [1,2,3,4]. The use of assisted reproductive techniques (ARTs) has led to a rise in monozygotic, and thus, monochorionic twinning. Among pregnancies achieved with ARTs, the incidence of monozygotic twins is reported to be around 2–5% [5,6,7,8,9]. It is chorionicity, rather than zygosity, that determines pregnancy outcomes. Establishing it correctly in the first trimester is crucial to adequately monitor the pregnancy and being able to recognize complications that are uniquely associated with the sharing of the common placenta. Among them, the most common is the twin-to-twin transfusion syndrome (TTTS) [2,10,11].

Approximately 10–15% of monochorionic pregnancies are complicated by TTTS [12]. The presence of arteriovenous placental vascular anastomoses that connect the two fetal circulations and lead to volume imbalance between the twins is considered to be the anatomical prerequisite of TTTS. Hypovolemia and subsequent oliguria on the donor twin on one side, and volume overload and polyuria on the recipient twin on the other side, lead to amniotic fluid disbalance between them [13,14,15].

The diagnosis of TTTS is done using sonographic criteria (oligohydramnios with deepest vertical pocket (DVP) < 2 cm, absent bladder for the donor twin; polyhydramnios defined as DVP > 8 cm prior to 20 weeks of gestation or >10 cm after 20 weeks of gestation, distended bladder for the recipient twin) [2,11]. The Quintero staging system has traditionally been used to grade the severity of the disease (Table 1) [16,17].

If left untreated, TTTS is associated with a high perinatal mortality rate, mainly due to spontaneous abortion and polyhydramnios-related preterm birth [3,18,19,20]. Furthermore, loss of one twin (single intrauterine death, sIUD) can lead to brain injury or death of the co-twin in around 26% and 15–20% of the cases, respectively, due to exsanguination of the surviving twin into the demised twin’s circulation through the vascular anastomoses [2,11,21,22,23].

Prior to the era of fetoscopic laser surgery, aggressive amnioreduction was used as a treatment option for TTTS with reported fetal survival rates ranging between 33% and 83% in the literature. The normalization of the amniotic fluid volume in the recipient’s sack is thought to reduce the risk of polyhydramnios-related spontaneous abortion and extremely premature delivery. Moreover, it is speculated that relief of polyhydramnios improves the uterine perfusion and the hemodynamic situation of the donor twin by releasing the hydrostatic pressure on its placental territory, thus slowing the natural progression of the disease [14,19,24]. Even though technically simple, the procedure carries the risk of complications, like placental abruption, rupture of membranes, chorioamnionitis, and chorioamniotic membrane separation [24,25]. Since amniondrainage does not directly address the cause of the problem, repeated procedures every one to two weeks are often necessary [19,24,25,26].

Fetoscopic laser occlusion of the communicating vessels as a treatment modality for monochorionic twin pregnancies complicated with severe previable TTTS was first described by Lia et al. in 1990. The rationale behind separating the two circulations is twofold. It lies in interrupting the transfusion between the twins, and also in protecting the surviving twin from exsanguination in case the co-twin dies in utero [24,27,28].

The aim of our study was to address the treatment options offered to patients with monochorionic diamniotic twin pregnancies complicated by TTTS in Germany, investigate the trajectory of each one of them through the past years, and discuss existing knowledge and controversies. Further, based on historical trends, we developed a prediction model that depicts future needs in fetoscopic laser surgery and addresses its implications for patients, healthcare providers, and healthcare systems in the years to come.

## 2. Materials and Methods

Data from the German Federal Statistical Office, including information about the annual number of surgeries for inpatients in the German Healthcare System between the years 2005 and 2021, were available for analysis. The operation and procedure classification codes (OPS Codes) related to fetal surgery were systematically examined. Two OPS Codes, corresponding to Fetal Laser Therapy (FLT) (2005–2021) and Serial Amniondrainage (SAD) (2006–2021), respectively, were identified as relevant to our research question. Even though the OPS Codes System undergoes annual updates, these two codes remained identical over the 16-year research period.

### 2.1. Statistical Analysis

#### 2.1.1. Trends Analysis in TTTS-Related FLT as a Percentage of Total Deliveries and Multiple Pregnancies

With regard to TTTS-related FLT surgery cases and in order to identify and analyze their trajectory over the study period, univariate linear regression analysis with time as an independent variable was performed using RStudio 2024.04.2. Results are presented as β-value, R^2^, *p*-value, and 95% confidence interval (CI). Two models were developed to examine the impact of the number of multiple pregnancies per year on one hand, and the number of deliveries per year, on the other hand on the trends of FLT surgeries over time.

Further, to detect possible age-related influences, linear regression models with the percentage of multiple pregnancies involving FLT as the dependent variable and time as the independent variable were built for the specific age groups. To determine the statistical significance in the age-specific percentage of multiple pregnancies involving FLT, an analysis of variance (ANOVA) was employed. Afterward, a post hoc analysis using Tukey’s honest significant difference (HSD) test was employed to determine which specific groups’ means were significantly different from each other.

A *p*-value of <0.05 was considered statistically significant.

#### 2.1.2. Cluster Analysis

The elbow test was applied to determine the optimal number of clusters. The “elbow point” is the point where the within-cluster sum of squares (WSS) starts to level off. It represents a balance between minimizing the WSS and avoiding overfitting with too many clusters. The variability within the clusters was determined by the standard deviation (SD).

The features selected for clustering were the total cases of FLT and the total number of deliveries. The selected features were standardized to ensure equal contribution to the clustering process. The K-Means clustering algorithm was used to identify distinct groups within the data [29]. K-Means partitions the data into K clusters, where each data point belongs to the cluster with the nearest mean. Each data point was assigned to one of the three clusters based on the nearest cluster center. The clusters were visualized using a scatter plot, with different colors representing different clusters. The centers of each cluster were calculated and transformed back to the original scale for interpretation.

#### 2.1.3. Calculation of Projection in FLT

Further, we aimed to address future FLT surgical needs. Historical data from 2005 to 2021 were used to make projections for future years. A linear regression analysis was used to predict the total number of deliveries for the years 2025, 2030, 2035, and 2040. The year, representing the time component and allowing us to observe the trend in the total number of deliveries over the years, was used as the independent variable (x) in the model. As the dependent variable (y), the total number of deliveries for each year was used. A linear relationship between the year and the total number of deliveries was presumed.

## 3. Results

In Germany, spanning the period from 2005 to 2021, 2952 TTTS-related surgical interventions were identified by their corresponding OPS Codes. Among them, 91.7% (n = 2706) accounted for FLT and 8.3% (n = 246) for SAD. The number of FLT surgeries ranged from 71 to 216 procedures per year, with a mean of approximately 159 procedures per year. The total number of deliveries ranged from 665,072 to 798,912, with a mean of approximately 723,350 deliveries per year. With regard to multiple pregnancies, their number ranged from 10,699 to 14,712, with a mean of approximately 12,642 per year.

### 3.1. Development of FLT and SAD in Germany over Time

**Figure 1:**
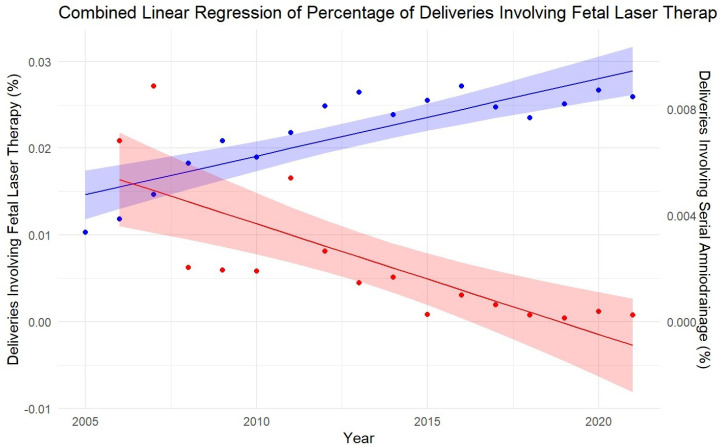
Development of fetal laser therapy and serial amniondrainage as a percentage of de-liveries in Germany between 2005 and 2021: The plot shows the linear regression line with the 95% confidence interval shaded around it. The left y-axis represents the percentage of deliveries involving fetal laser therapy surgeries (FLT, blue line), and the right y-axis represents the percentage of deliveries involving serial amniondrainage (SAD, red line). The linear regression line, along with the 95% confidence interval shaded around it, illustrates the statistical significance of the increasing use of FLT and the decreasing trend in SAD.

Over the study period, a statistically significant increase in the number of FLT surgeries and a noteworthy decline in the number of SAD procedures were noted. The univariate linear regression analysis revealed a significant increase in the percentage of FLT cases with respect to both the number of deliveries (β = 0.00089; *p* < 0.001; and R^2^ = 0.73), (Figure 1) and the number of multiple pregnancies (β = 0.042; *p* < 0.001; and R^2^ = 0.69). Concomitantly, a significant decrease in numbers and percentage of deliveries involving SAD (Figure 1), and percentage of multiple pregnancies involving SAD was detected.

### 3.2. FLT and Maternal Age

**Figure 2:**
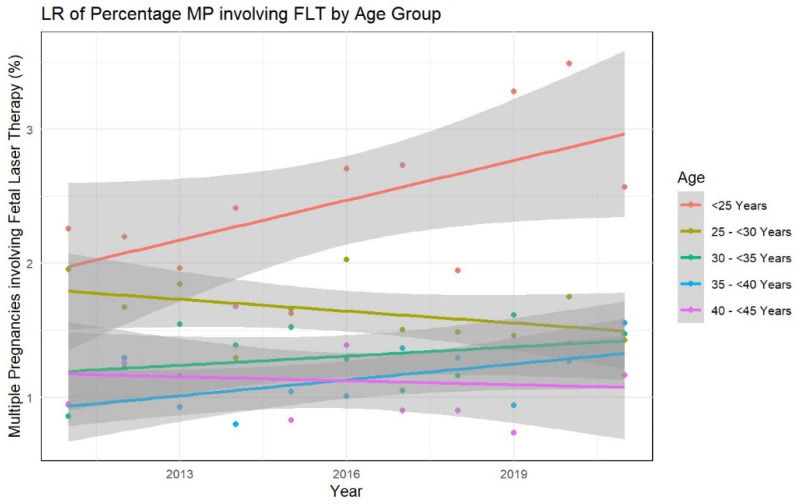
Percentages of multiple pregnancies involving fetal laser therapy surgery by maternal age groups over time: The plot includes the regression lines with 95% confidence intervals for each age group. The x-axis represents the years, and the y-axis represents the percentage of multiple pregnancies involving fetal laser therapy surgery between 2011 and 2021. Each color corresponds to a different age group. The regression lines and 95% confidence intervals for each age group are plotted to show the trend in the use of FLT across different maternal age categories. LR: likelihood ratio, MP: multiple pregnancies, and FLT: fetal laser therapy.

To assess maternal age-dependent variations in the development of the use of FLT as a percentage of multiple pregnancies over time, we performed a linear regression analysis with time as the independent variable. The results are visualized in Figure 2. As very few cases occurred in the age group > 45 years, we withdrew that specific age group from the analysis. While the age groups < 25 years, 30– < 35 years, and 35– < 40 years showed a positive trend in the linear regression model with time as an independent variable, none of the age groups showed statistical significance (all *p*-values > 0.05).

To assess if there were any significant differences between the percentages of multiple pregnancies involving FLT between different age groups, we performed an ANOVA analysis, which proved to be statistically significant (df = 4, F = 33.13, *p*-value < 0.001). The comparison between age groups performed by the post-hoc Tukeys HSD test showed that the “<25 years” group had significantly higher percentages compared to all other age groups (25– < 30; 30– < 35; 35– < 40; and 40– < 45 years).

### 3.3. Clusters of Development of Fetal Laser Therapy over Time in Germany

By using the elbow test, we determined three clusters (cluster 1: 2005–2007; cluster 2: 2008–2014; and cluster 3: 2015–2021) that were optimal to describe the development of FLT in Germany over the years. The cluster characteristics are shown in Table 2.

**Figure 3:**
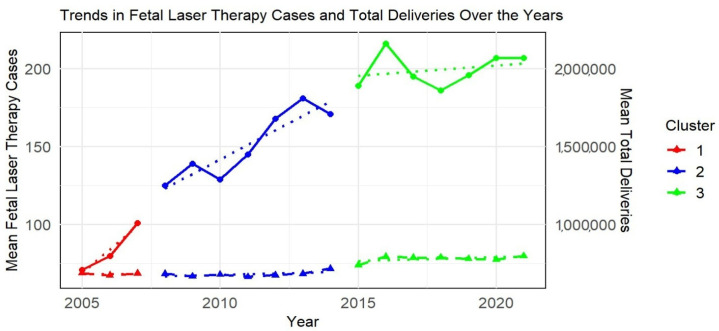
Clusters of development in fetal laser therapy in Germany from 2005 to 2021: Scatter plot visualizing the clustering of fetal laser therapy (FLT) surgeries across three distinct periods (Clusters 1, 2, and 3), with each cluster representing a different phase in the development of FLT in Germany. The solid lines represent FLT surgery cases, while the dashed lines show total deliveries (scaled down by 10,000). The points on the plot represent the mean values, and the triangles highlight the center of each cluster. Cluster 1: years 2005–2007; cluster 2: years 2008–2014; cluster 3: years 2015–2021, solid lines: fetal laser therapy surgery cases, dashed lines: total deliveries (scaled down by 10,000), points: mean values, triangles: mean values, and dotted lines: trend lines.

The clusters represent three different periods in the dataset, each with distinct characteristics in terms of FLT cases, total deliveries, and women with multiple pregnancies (Table 2). The visualization plot (Figure 3) showed a clear separation between the clusters, indicating distinct groups based on the number of FLT cases and total deliveries. In terms of FLT, cluster 3 presented a significantly higher mean number (199.43) compared to cluster 2 (151.14) and cluster 1 (84.00). Cluster 3 (SD = 10.88) showed a decreased number in variability than cluster 2 (SD = 22.09) and cluster 1 (SD = 15.39).

### 3.4. Future Trends in FLT

In order to project future trends in FLT, we used the available historical data from 2005 to 2021 and applied linear regression models based on the percentage of total pregnancies and multiple pregnancies involving FLT. Based on these two models, we assume a rise in FLT surgeries, as shown in Table 3.

## 4. Discussion

### 4.1. Development of FLT and SAD in Germany and Beyond over Time

To our knowledge, this is the first analysis of trends in TTTS treatment strategies based on an inpatient nationwide dataset in Germany spanning 16 years of observation. In this retrospective analysis of the evolution of TTTS treatment strategies, we observed a significant decrease in SAD procedures and an increase in the use of FLT in Germany, both in terms of total deliveries and multiple pregnancies per year. The *p*-values for both models indicate that these trends are statistically significant (*p* < 0.001 for both). The increase in the percentage of total deliveries involving subsequent FLT provided a slightly better fit, with a higher R-squared value (0.73) compared to the percentage of multiple pregnancies with subsequent FLT (0.69). This suggests that the trend related to total deliveries explains more of the variability in the data.

The linear regression model for the dependent variable “percentage of multiple pregnancies involving FLT” presented a much higher β-value, indicating a stronger annual increase in the percentage of FLT cases with respect to multiple pregnancies (β = 0.042) than to total deliveries (β = 0.00089). This phenomenon may be attributed to the fact that the incidence of multiple pregnancies is disproportionately increasing, driven by factors such as advancing maternal age and the widespread utilization of assisted reproductive techniques (ART) [30].

This shift in TTTS treatment strategies is closely linked to the evolution of the fetoscopic laser technique on one side and increased awareness for the condition by obstetricians performing antenatal exams for monochorionic twin pregnancies on the other side. Increased awareness for early diagnosis and appropriate case selection for surgery, followed by prompt intervention and prolongation of pregnancy through the optimization of the surgical technique and targeted follow-up in order to detect and manage postoperative complications are important factors that have likely contributed to improved short- and long-term out-comes for TTTS survivors. [15,31,32,33,34,35,36]. In Germany, the first FLT for TTTS was performed in the Department of Prenatal Diagnosis and Therapy, Barmbek Hospital, Hamburg, Germany in January 1995. The surgeons evaluated and reported their results and showed improved survival rates with growing experience and implementation of the selective laser coagulation technique [1,37].

Several studies aimed to compare FLT with SAD in terms of survival rates and neurologic morbidity rates for the treatment of TTTS. The Eurofoetus trial was a multicenter, randomized trial that clearly showed the superiority of FLT [17]. Subsequent studies confirmed these findings, showing the benefits of FLT in terms of survival rates, neurologic outcome, and gestational age at delivery [1,38,39,40]. With regard to long-term neurologic outcome, the review of the literature reveals that both twins are equally at risk of suffering cerebral injury [41,42], whereas FLT-treated TTTS survivors are at reduced risk of long-term major neurodevelopmental impairment compared to those treated with SAD [43,44,45].

The optimal timing for initiating fetal therapy still remains unclear [46]. Even though universally used, the Quintero Staging system is not a good predictor of outcome. The Quintero staging system does not modify the definition of polyhydramnios with advancing gestational age. The Eurofoetus trial considered a deepest vertical pocket of >10 cm for the definition of polyhydramnios in pregnancies after 20 weeks of gestation [17]. Moreover, disease progression does not necessarily follow a continuum through the Quintero stages and fetal cardiac functional assessment is not incorporated into the Quintero staging system. However spontaneous fetal demise and cardiovascular dysfunction of the recipient twin may occur even as early as in stage I and II TTTS disease [47,48,49]. The strongest argument for offering immediate surgery is the protection of the surviving twin in case the co-twin dies in utero. The benefits of early intervention need to be balanced with the surgical risks (premature rupture of membranes, chorioamnionitis, placental abruption, fetal-maternal hemorrhage, iatrogenic monoamnionicity, post-laser TAPS, and recurrent TTTS) and the risk of natural progression in higher disease stages [47,48,50]. At least for TTTS stage I, expectant management with heightened surveillance seems to be a reasonable option.

### 4.2. FLT and Maternal Age

To our knowledge, we showed, for the first time, that the percentage of multiple pregnancies involving FLT significantly varies across maternal age groups. In contrast to other pregnancy-related complications, such as fetal growth restriction, preeclampsia, stillbirth, and gestational diabetes mellitus, which increase with maternal age, in our study population, the risk of multiple pregnancies requiring FLT was significantly higher in younger mothers under 25 years of age, than in all other age groups [51].

One possible explanation for this phenomenon might be a lower risk of vanishing twin in pregnancies of women <25 years, for example as a result of a higher frequency of correct cleavage of the embryo, leading to more monochorionic pregnancies reaching the second trimester, and consequently, more cases requiring FLT in this age group. The relationship between maternal age and monozygotic (MZ) twinning remains incompletely understood, with conflicting evidence in the literature. While some studies suggest advanced maternal age may reduce MZT rates in assisted reproductive technology (ART) cycles [5,52], others report no significant association in spontaneous pregnancies [53]. In our cohort, the higher frequency of FLT in women <25 years could reflect age-related biological factors influencing spontaneous MZ twinning rates, though the exact mechanisms are unclear. Previous ART studies indicate younger oocyte age increases MZT risk [5], but this does not fully explain our findings given the low utilization of IVF in this age group. The observed pattern may suggest intrinsic differences in early embryonic development across maternal ages, though current evidence remains insufficient to establish causation. Further research is needed to elucidate whether maternal age independently affects embryogenesis processes leading to monochorionic twinning in natural conceptions.

Comparing our results with previous reports in the literature is difficult, since sociodemographic variables in fetal surgery studies are quite underreported. A recently published systematic review by Wilpers et al. revealed that only 33% of the 112 included fetal surgery studies reported on maternal age. In this systematic review, TTTS represented the most common disease group [54]. Future studies are needed to confirm our findings. From our point of view, efforts need to be made to address the critical gaps in sociodemographic reporting in fetal surgery studies.

### 4.3. Cluster Analysis

Cluster analysis in healthcare is essential for identifying patterns, detecting anomalies, segmenting data, and informing resource allocation and decision-making, thereby enhancing the understanding and management of medical procedures and rare conditions [55,56,57]. These factors are critically important as healthcare systems in advanced economies, like Germany, and globally, face the challenge of integrating rapidly evolving medical technologies to provide optimal evidence-based healthcare. Concurrently, they must manage healthcare expenditures to maintain sustainable contribution limits and ensure financial stability within the system [58].

Using the K-means algorithm for cluster analyses, an informative and easily interpretable method of clustering, we identified three distinct periods (2005–2007, 2008–2014; 2015–2021) in the development of FLT in Germany [57]. Cluster 3, representing the period from 2015 to 2021, exhibited a significantly higher mean number of FLT cases compared to clusters 1 and 2, while demonstrating lower variability, indicating a more consistent number of FLT cases in recent years. In contrast, the number of FLT cases in cluster 2 showed greater variability. Cluster 2 represented the transitional phase of FLT, from its introduction and initial implementation in cluster 1 to its standardization in cluster 3. Both clusters 2 and, more pronouncedly, cluster 3, displayed an increasing trend in the number of FLT, reflecting overall growth and increased adoption of FLT as a sign of the implementation and standardization of treatment.

The observed increase in FLT procedures over time (cluster 2 and cluster 3) may reflect, in part, heightened awareness and evolving clinical practice. However, our results do not include direct measures of advancements in medical technology, patient counselling, or rates of procedure acceptance versus declination. Thus, we cannot take definitive conclusions regarding the drivers in increase of FLT adoption during the identified cluster periods. Additionally, as FLT is covered by healthcare insurance, and the majority of the population is insured, reimbursement by insurance is unlikely to have impacted the increase in the number of FLT cases [58].

Assuming that the historical trend for an increase in the number of deliveries observed from 2005 to 2021 will continue linearly, the linear regression analysis revealed an increasing number of FLT in future years (2025, 2030, 2035, and 2040). Projections of future numbers of FLT are critical for healthcare resource allocation and planning within the medical field. Given that FLT is typically employed for specific and relatively rare conditions, the frequency of this procedure is considerably lower compared to other prenatal care interventions. The lower incidence of these conditions makes projections more sensitive to minor variations in data, thereby complicating the accuracy of such predictions. While projections may never achieve 100% accuracy, they are crucial for informing insurance and healthcare policies.

According to the evidence gained by the cluster analysis, we expect an increase in the number of FLT surgeries in the near future, but at a lower rate than in the years 2005–2007 and 2008–2014. These projections address future needs, such as an increase in the number of specialized care centers and highly specialized medical teams performing this procedure. Apart from the required infrastructure, including facilities and equipment, there is a great need to train maternal–fetal medicine specialists to be able to accommodate the rising number of cases. The first step before any intervention can be initiated is to establish the correct diagnosis and properly grade the severity of the disease. This requires training in prenatal ultrasonography and heightened awareness among primary healthcare providers, since early diagnosis and timely intervention, when indicated, are crucial in order to improve pregnancy outcomes. Adequate perinatal settings are also crucial in order to ensure the postoperative follow-up of the treated cases and management of potential surgery-related complications [3].

The next step is to be able to perform fetal surgery safely. In order to do so, a learning process requiring hands-on training is essential, especially since increasing operating experience and caseloads are important factors influencing perinatal outcomes [37,39,59,60]. Given that the disease is rare and that FLT is only performed in specialized centers worldwide, training the next generation of fetoscopic surgeons poses great challenges.

### 4.4. Strengths and Limitations

Key strengths of this study lie in its methodologic rigor and its large sample size, especially given the rarity of TTTS. This study benefits from a large-scale, national dataset spanning 16 years (2005–2021), providing a comprehensive overview of trends in TTTS-related procedures (FLT and SAD) in Germany. The large sample size lends robustness to the observed trends and projections.

Our study has major implications for health care systems and health care providers. Our results underscore the need for both enhanced training and effective resource allocation in order to ensure that the necessary infrastructure is in place to support the growing demand for fetoscopic laser surgery.

Nevertheless, some limitations must be acknowledged. This study relies on data derived from the German Federal Statistical Office. Unfortunately, the data do not include detailed clinical parameters, such as gestational age at diagnosis, time interval between diagnosis and treatment, TTTS severity, or the specific type of laser therapy used (selective vs. non-selective). These missing clinical data prevent a deeper analysis of treatment outcomes or a comparison of effectiveness between different approaches. Additionally, the dataset does not capture clinical outcomes, such as perinatal and neonatal survival rates and neurologic morbidity rates. This limits the ability to draw direct conclusions about the effectiveness of FLT versus SAD and to assess the long-term outcome of TTTS survivors.

Another limitation is that the prediction model, developed to assess future trends, considers only the year as the predictor. Other potential factors that could influence the number of deliveries, such as demographic changes, healthcare policies, economic conditions, and social factors, were not included in this simple linear regression model. The projections provided are based on the assumption that the historical trend observed from 2005 to 2021 will continue linearly into the future. While this provides a straightforward method for making projections, it is important to consider that real-world trends can be influenced by a variety of factors not captured in this simple model. Despite these limitations, healthcare policymakers can utilize these insights to formulate strategies that address the rising demand for fetal laser surgery and improve maternal and fetal health outcomes.

The results of this study are only applicable to the German healthcare system and are not generalizable to other high-level healthcare systems, since prenatal care and, thus, prenatal counseling, are not only rooted in evidence-based medicine results but also in traditions and may differ across cultures [58].

## 5. Conclusions

Advances in prenatal care have led to a substantial improvement in pregnancy and neonatal outcomes. Still, only 85% of monochorionic pregnancies diagnosed in the first trimester result in two liveborn twins. TTTS is the most common complication associated with the sharing of the common placenta. Endoscopic laser coagulation of the anastomotic vessels poses the only, for the moment, direct and causative treatment of the disease and is considered the best treatment option for TTTS stages II and above.

To our knowledge, an association between the need for fetoscopic laser surgery for TTTS and maternal age has not previously been described in this context. In our cohort, the percentage of multiple pregnancies requiring FLT was higher in younger mothers aged <25 years. Until now, maternal age has been quite underreported, making a comparison of our results with previous fetal surgery studies impossible. Further studies are needed to confirm our findings.

The results of our analysis reveal a notable increase in the number of fetoscopic laser surgical procedures in the past years in Germany. The same applies to the projections for the future years. This increasing demand presents a great challenge for healthcare providers and healthcare systems. Despite its clear life-saving benefits, the accessibility of fetoscopic laser surgery is still limited, since the procedure is offered at only specialized fetal therapy centers nationwide. A lot more needs to be done to make fetoscopic laser surgery accessible and safe for all pregnant women. From our point of view, it is essential to address the disparities in access to fetal laser therapy and prenatal care, and ensure that all women, regardless of socioeconomic status or geographic location, receive the care they need. Geographical and socioeconomic barriers need to be overcome and time intervals between diagnosis and treatment need to be reduced.

Our study makes clear that future focus should lie on allocating resources efficiently in order to ensure that the necessary infrastructure is in place to support the growing demand for FLT. This encompasses developing and implementing comprehensive surgical training curricula on one hand, and ongoing reporting and monitoring of outcomes in order to ensure satisfactory results on the other hand.

## Figures and Tables

**Table 1:** Quintero staging system for TTTS.

Stage	Classification
I	Polyhydramnios in recipient twin with DVP > 8 cm before and >10 cm after 20 weeks of gestation Oligohydramnios in donor twin with DVP < 2 cm
II	Urinary bladder of donor twin not visible on ultrasound
III	Doppler velocimetry abnormalities in either twin: - Absent or reversed umbilical artery diastolic flow - Ductus venosus with negative a-wave - Pulsatile umbilical venous flow
IV	Hydrops in one or both twins
V	Demise of one or both twins

DVP: deepest vertical pocket; cm: centimeter.

**Table 2:** Characteristics of the three clusters of development in fetal laser therapy surgery in Germany between 2005 and 2021.

Cluster	Mean_ FLT	Range_ FLT	SD_ FLT	Mean_ Total_ Deliveries	Range_ Total_ Deliveries	SD_ Total_ Deliveries	Mean_ Multiple_ Pregnancies	Range_ Multiple_ Pregnancies	SD_ Multiple_ Pregnancies
1	84	30	15.39	683,553	13,138	7301.27	143.33	66	33.50
2	151.14	56	22.09	682,281.14	52,452	17,387.25	172.43	52	19.07
3	199.43	30	10.88	781,475.43	58,550	19,700.30	154.29	77	26.05

FLT: fetal laser therapy surgery, cluster 1: years 2005–2007; cluster 2: years 2008–2014; cluster 3: years 2015–2021, and SD: standard deviation.

**Table 3:** Projections in fetal laser therapy surgeries in Germany as a percentage of total deliveries and percentage of multiple pregnancies.

Year	Total_ Predicted	Total_ Lower_CI	Total_ Upper_CI	Multiple_ Predicted	Multple_ Lower_CI	Multiple_ Upper_CI
2025	0.033	0.03	0.04	1.755	1.55	1.95
2030	0.03	0.03	0.04	1.96	1.69	2.23
2035	0.04	0.03	0.05	2.17	1.83	2.52
2040	0.05	0.04	0.05	2.39	1.97	2.80

CI: confidence interval.

## Data Availability

Access to the raw data can be provided upon reasonable request to the corresponding author.

## Abbreviations

The following abbreviations are used in this manuscript:

TTTS:	Twin-to-twin transfusion syndrome
FLT	Fetal laser therapy
SAD	Serial amniondrainage
ART	Assisted reproductive techniques
DVP	Deepest vertical pocket
sIUD	Single intrauterine death
OPS	Operation and procedure classification codes
CI	Confidence interval
ANOVA	Analysis of variance
HSD	Honest significant difference
WSS	Within-cluster sum of squares
SD	Standard deviation
LR	Likelihood ratio
MP	Multiple pregnancies
TAPS	Twin anemia–polycythemia sequence
IVF	In vitro fertilization
MZT	Monozygotic twinning
